# 3D Tomographic Analysis of the Order‐Disorder Interplay in the *Pachyrhynchus congestus mirabilis* Weevil

**DOI:** 10.1002/advs.202202145

**Published:** 2022-07-18

**Authors:** Kenza Djeghdi, Ullrich Steiner, Bodo D. Wilts

**Affiliations:** ^1^ Adolphe Merkle Institute University of Fribourg Chemin des Verdiers 4 Fribourg 1700 Switzerland; ^2^ Chemistry and Physics of Materials University of Salzburg Jakob‐Haringer‐Straße 2a Salzburg 5020 Austria

**Keywords:** 3D structure characterization, biophotonics, disorder, FIB‐tomography, Pt backfilling

## Abstract

The bright colors of *Pachyrhynchus* weevils originate from complex dielectric nanostructures within their elytral scales. In contrast to previous work exhibiting highly ordered single‐network diamond‐type photonic crystals, here, it is shown by combining optical microscopy and spectroscopy measurements with 3D focused ion beam (FIB) tomography that the blue scales of *P. congestus mirabilis* differ from that of an ordered diamond structure. Through the use of FIB tomography on elytral scales filled with platinum (Pt) by electron beam‐assisted deposition, it is revealed that the red scales of this weevil possess a periodic diamond structure, while the network morphology of the blue scales exhibit diamond morphology only on the single scattering unit level with disorder on longer length scales. Full wave simulations performed on the reconstructed volumes indicate that this local order is sufficient to open a partial photonic bandgap even at low dielectric constant contrast between chitin and air in the absence of long‐range or translational order. The observation of disordered and ordered photonic crystals within a single organism opens up interesting questions on the cellular origin of coloration and studies on bio‐inspired replication of angle‐independent colors.

## Graphical abstract

Interference in structurally ordered materials gives rise to brilliant colors in nature while disorder is thought to destroy color. Yet, many insects employ quasi‐ordered structures to generate brilliant colors. Here, the order–disorder interplay in the biophotonic materials of an Easter Egg weevil is demonstrated through 3D electron‐microscopy‐based imaging combined with optical modeling.

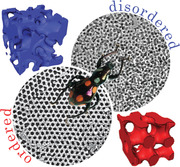

## 1 Introduction

Color in nature can be of pigmentary and/or structural origin, with pigmentary color resulting from the absorption of specific wavelengths of light by chemical substances and structural color resulting from the scattering of incident light by periodic nanostructures. Numerous organisms rely on structural color, either on its own or coupled with pigments, to provide crucial survival functions including aposematism, mating, or camouflage.^[^ ^1^, ^2^ ^]^ The advantage of structural over pigmentary coloration is their brightness, angle‐dependence (iridescence) and/or polarization‐dependence, which are inaccessible by pigments alone.^[^ ^3^, ^4^, ^5^ ^]^ A variety of photonic structures with increasing structural complexities have been identified in natural systems. These include low‐dimensional periodic systems in the form of simple thin films or multilayers in insect wing scales and elytra,^[^ ^6^ ^]^ diffraction gratings in flowers,^[^ ^7^ ^]^ and more complex 3D periodic sub‐micron structures, which can for example be found in beetle, weevil and butterfly scales.^[^ ^8^, ^9^, ^10^ ^]^ These periodically ordered dielectric nanostructures are known as photonic crystals (PCs). Since the building blocks of PCs are positioned on a regular lattice, the structures possess both long‐ and short‐range order. If the periodicity is on a length scale enabling interference in the visible spectrum and provided the refractive index (RI) contrast between the different phases is sufficiently high, PCs open a complete or partial bandgap resulting in structural coloration.^[^ ^11^, ^12^, ^13^ ^]^

By contrast, amorphous networks lack both short‐ and long‐range order. In these networks, structural parameters such as the connectivity, nearest neighbor distance, and bond angles are randomly distributed. Optical amorphous networks are highly diffusive due to the multiple scattering of light with little correlation in the phase or directionality of individual ray trajectories, resulting in white coloration.^[^ ^14^ ^]^ Several species of *Coleoptera* rely on this principle to produce a striking white “color”.^[^ ^15^, ^16^, ^17^ ^]^ Between perfect order and complete disorder, quasi‐ordered structures possess local short‐range correlations and suppressed long‐range fluctuations, while lacking long‐range translational order. This encompasses, for instance, poly‐crystals with small‐sized domains, continuous random networks (CRN) with fixed valency, hyperuniform structures, and quasi‐crystals.^[^ ^18^, ^19^, ^20^ ^]^ In some special cases, the spatial correlations, which are on a scale comparable to optical wavelengths, are sufficient to generate partial bandgaps and hence structural coloration. The produced color is more diffuse, less saturated, and angle‐independent compared to colors produced by highly ordered PCs. Partially disordered bicontinuous structures are a less common mechanism to generate color in nature and observations have so far been limited to blue coloration, mainly in beetle scales and bird feathers.^[^ ^21^, ^22^, ^23^, ^24^, ^25^, ^26^, ^27^, ^28^ ^]^

So far, studies of amorphous structures in biological systems have predominantly relied on 2D data collection from cross‐sectional electron microscopy,^[^ ^21^, ^22^, ^25^ ^]^ or small‐angle X‐ray scattering studies.^[^ ^29^ ^]^ While both methods enable the identification of periodic morphologies, an imaging approach that elucidates the full 3D structure is required to fully understand the interplay between the structure and the optical signature of non‐periodic photonic materials.^[^ ^27^ ^]^

In this work, we examine the full 3D structure in the elytral scales of the *Pachyrhynchus congestus mirabilis* weevil and link it to the optical appearance of the scales. Previous work on *Pachyrhynchus* weevils has discussed either single colored spots or rainbow‐colored spots on jet‐black elytra.^[^ ^9^, ^30^, ^31^ ^]^ These studies found an ordered single‐network diamond structure (space group $Fd\bar{3}m$) in their scales,^[^ ^30^, ^31^ ^]^ with color variations resulting from changes in the lattice constant, chitin filling fraction and/or domain orientation. Through focused ion beam scanning electron microscopy (FIB‐SEM) 3D reconstructions of the photonic networks within the wing scales of *P. c. mirabilis* and by using a Pt‐based deposition technique, this study demonstrates the origin of color from ordered and seemingly amorphous PC structures. Comparing optical experiments with full wave 3D simulations of the 3D datasets combined with a 3D structural analysis allows insights into insects' coloration mechanisms and provides interesting further avenues to investigate the developmental cellular pathways of insect nanostructures,^[^ ^10^ ^]^ but also the use of amorphous diamond‐based structures as photonic materials.^[^ ^32^ ^]^

## 2 Results and Discussion

### 2.1 Optical Appearance and Anatomy

**Figure 1:**
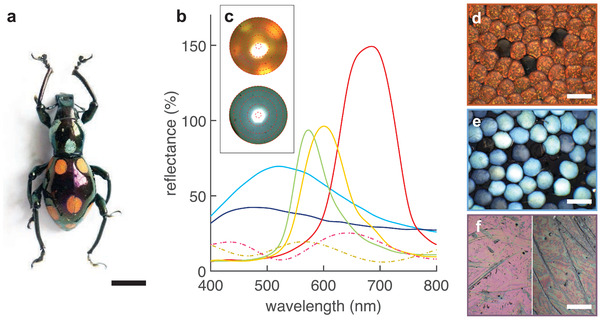
a) Photograph of a *P. c. mirabilis*. b) Representative reflectance spectra of the elytron and scales from different regions measured with a microspectrophotometer (*n* = 1 for each curve). The blue patches contain single‐colored bright and dull blue scales giving rise to single broad reflectance peaks with maxima under 520 nm, while red patches contain red scales comprising a majority of red domains as well as a minority of local yellow‐, and green‐ colored domains giving rise to more narrow single reflectance peaks with maxima between 575 and 700 nm. Reflectance values over 100% occur as a white diffuser served as a reference. The curves are plotted in a color approximately matching the observed color and have been smoothed to remove experimental noise. The dashed lines correspond to the reflectance of the elytron. c) Scatterograms of the red (top) and blue (bottom) scales. The red‐dashed circles represent scattering angles of 5°, 20°, 35°, 50°,  and 64°  from the center to the perimeter. d–f) Microscopy images of the various colored areas on the elytron: d) red scales, e) blue scales, f) abdomen (left) and thorax (right) elytron. Some transparent scales—that appear black due to the black‐colored underlying elytron—can also be observed. Scale bars: (a) 0.5 cm, (d–f) 100 µm.

*P. c. mirabilis* is an about 2 cm long weevil with a dark‐purple elytron that features red‐orange and blue colored ellipsoidal spots on its dorsal and lateral sides (**Figure** **1**a). A variable number of red‐orange spots are located exclusively on the abdomen and three blue spots are located on the thorax. Each spot is assembled of brightly colored oval scales with a mean long diameter of 80 ± 4 µm and a short diameter of 69 ± 4 µm, giving an average aspect ratio of 1.16 (*n* = 40). Visual inspection at varying angles revealed a pink glare of the elytron (Figure 1a,f). Although they appear uniformly colored to the naked eye, optical microscopy observations reveal that the red‐orange scales contain multiple domains that vary in color (Figure 1d) from green to red. In contrast, the hue of the blue scales varies, with the darker ones appearing more dull but remain uniform in color across each individual scale (Figure 1e). Note that both blue and red spots also contain a few transparent scales, which are not discussed here.

To quantify the coloration and investigate its origin, we measured reflectance spectra of individual elytral scales attached to the elytron with a microspectrophotometer. Representative reflectance spectra are shown in Figure 1b. The scales from the red‐orange spots yielded narrow single peaks with full width at half maximum (FWHM) values between 70–110 nm centered around ≈575, 600, and 690 nm, corresponding to green, yellow and red facets, respectively. The amplitude of the reflection peaks increased with the peak wavelength. In contrast, the dull to bright blue scales gave rise to very broad single‐peaked spectra with FWHM values of ≈220 nm and reflectance peaks located between 470 and 515 nm. The presence of pigments was also investigated since they are common in natural systems and are often used in combination with organismal structural colors, as observed in beetle^[^ ^24^ ^]^ and butterfly^[^ ^10^ ^]^ scales or bird feathers.^[^ ^4^, ^5^ ^]^ Transmission spectra of both blue and red scales immersed in refractive index‐matching oil (*n*_o_ = 1.56, Figure S3, Supporting Information) indicate the presence of a broad‐band absorbing pigment that gradually increases in absorbance toward short wavelengths, reminiscent of melanin.^[^ ^33^ ^]^ This broad‐band absorbance however does not mirror the reflectance spectra and we can therefore ascribe the coloration to a combination of structural and pigmentary origins.

In addition, reflection spectra of the elytron displayed two low‐intensity reflection broad peaks characteristic of thin film interference, but the position of these peaks were different on the thorax and on the abdomen. The thorax, which bears the blue scales, appeared more black to the naked eye and displayed two peaks, one below 400 nm ($r_{\mathrm{avg}}=21\%$) and one at 565 nm (FWHM_avg_ = 130 nm, $r_{\mathrm{avg}}=16\%$). The abdomen, which carries the red scales, appeared more pink and displayed two peaks at 430 nm (FWHM_avg_ = 90 nm, $r_{\mathrm{avg}}=18\%$) and 640 nm (FWHM_avg_ = 140 nm, $r_{\mathrm{avg}}=22\%$).

To study the angle‐dependence of the scale coloration, *k*‐space measurements were performed by inserting a Bertrand lens into the imaging pathway (see Experimental Section). Figure 1c shows scatterograms taken with a narrow‐angle illumination for each type of elytral scale. The area of illumination covered several domains of the red scales and revealed directionally‐reflected spots of red, yellow and green visible at various angular positions. Quite differently, the blue scales showed uniform, broad‐angle scattering profiles, independent of their brightness, which are in contrast to the highly directional, narrow, high reflection peaks of the red scales. We hypothesize that these contrasting optical behaviors are the result of structural differences present within each type of scale.

### 2.2 Internal Structure of the Scales

**Figure 2:**
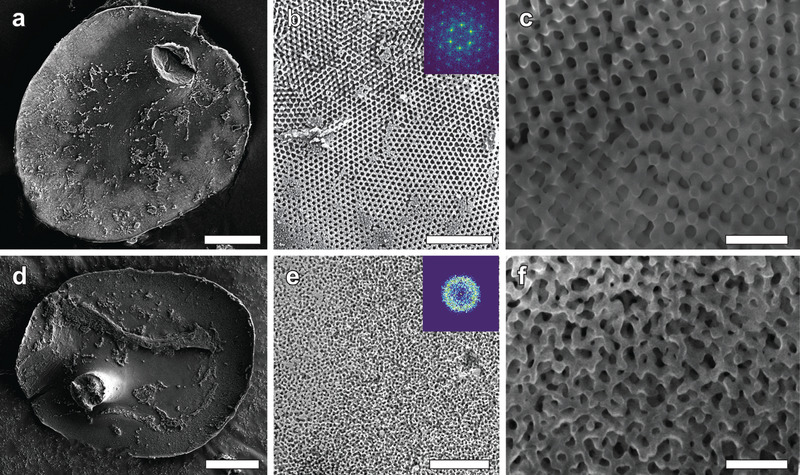
SEM images of a,d) whole scales, b,e) top views of cortex‐free regions of the scales, and c,f) cross‐sectional views created by FIB milling of red (a–c) and blue scales (d–f). The red scales feature a highly organized internal structure with hexagonal symmetry, while blue scales feature an amorphous chitin structure. The insets in (b,e) show 2D fast Fourier transforms of small areas of each image to highlight local order. Scale bars: (a,d) 20 µm, (b,e) 4 µm, (c,f) 1 µm.

To confirm the structural origin of the coloration and identify structural variations, top‐ and side‐view images of each type of scale were taken using a scanning electron microscope (SEM). For the side‐view cross‐sectional images, scales were cut using a focused Ga‐ion beam (FIB, see details in Experimental Section). **Figure** **2** shows the resulting overview images.

The interior of the red scales features an ordered photonic crystal with multiple domains (Figure 2b,c). Figure 2b shows a hexagonal chitin pattern in the plane perpendicular to the normal of the scale which is characteristic of a cubic structure. The diameter of large domains reaches approximately 55 µm^2^. The 2D FFT of this structure exhibits a clear sixfold symmetry with a periodicity of ≈300 nm (inset in Figure 2b). Polycrystalline diamonds are common in beetles scales and have been observed through a wide range of species to generate various colors.^[^ ^8^, ^9^, ^23^, ^34^ ^]^ In particular, these results are consistent with previous work on *Pachyrhynchus* weevil scales, where the structure within these scales was identified as a single‐diamond network of chitin and air.^[^ ^30^, ^31^ ^]^ The main parameters that influence the observed color of biological 3DPCs are i) its local orientation, ii) its lattice parameter and iii) its solid filling fraction.^[^ ^13^, ^30^ ^]^ Here, the 〈111〉 orientation is clearly dominant along the surface normal, but in‐plane rotations are visible, giving rise to the tessellated appearance of the scales (Figure 1d). Assuming a 〈111〉 orientation of a single diamond, the lattice parameter of the resulting network has a constant value of *a* = 489 ± 5 nm calculated from the average pore nearest neighbor distance in the (111) plane, *d*_nn_ = 346 ± 7 nm (Figure 2b). From the measured chitin area fraction of the (111) diamond plane, using the empirical formula ${\mathrm{ff}}_{3D}=\left(1.73\;\cdot {\mathrm{ff}}_{2D,111}\right)-0.83$, the estimated 3D filling fraction is ≈42%. These values are in line with previously reported data on the orange‐red scales of the *P. c. pavonius*, which had an estimated fill fraction of 36% and a lattice parameter of 483 nm.^[^ ^30^ ^]^

SEM imaging of the blue scales also shows a network of chitin in air, but no clear periodicity is visible (Figure 2e,f). A spatial frequency was however determined by 2D FFT, which yielded a diffuse disk with a pronounced ring indicating a preferred nearest neighbor distance which is spatially not very well defined (Figure 2e, inset). This length corresponds to the average distance between two pores of ≈250 nm. Since all blue scales, whether dull or bright, display similar spectral and scattering behaviors, and since observations through an electron microscope showed no clear variation from one blue scale to another, the remainder of the study focuses on a single representative blue scale.

### 2.3 3D Imaging using FIB‐SEM Tomography

3D data is needed to unambiguously characterize the scale structures and relate these to their photonic properties. So far, data of biophotonic structures collected by SEM or TEM imaging were often limited to 2D information.^[^ ^9^, ^21^, ^22^, ^24^, ^25^, ^35^ ^]^ Moreover, the process of obtaining a single cross‐section from a biological sample through TEM involves lengthy sample preparation that consists of embedding, staining and lift‐out, sometimes followed by demanding post‐processing to deconvolute the resulting image.^[^ ^36^, ^37^ ^]^ These methods are often “blind” so that a desired location within a 3D morphology cannot be extracted.

Numerous studies have used small‐angle X‐ray scattering (SAXS) to gather 3D information from a wide range of biophotonic samples.^[^ ^26^, ^29^, ^38^ ^]^ For ordered systems, SAXS often enables space group assignment as well as quantification of structural parameters. The SAXS results for disordered structures are less clear and show a diffuse disk for amorphous structures, while quasi‐ordered structures feature a pronounced ring from which short‐range spatial periodicity can be extracted. The assignment of a full 3D morphology using SAXS is however not always unambiguous and can benefit from complementary real‐space imaging techniques. Imaging is especially beneficial in the case of amorphous and quasi‐ordered systems, as it allows direct visualization of the 3D arrangement of matter in a chosen region of interest, while SAXS usually averages over a larger volume and does not give information on local arrangements. 3D imaging on this length scale (10 nm–1 µm) can also be achieved through ptychographic X‐ray‐based tomography.^[^ ^15^ ^]^ Both SAXS and X‐ray tomography however require access to a synchrotron with distinct beamline configurations or specialized lab equipment.^[^ ^10^ ^]^ Real‐space tomography techniques are a good alternative/complementary technique to consider. In particular, FIB‐SEM tomography datasets can be automatically acquired in a few hours and require very little ex situ preparation, while allowing for the extraction of a very large set of cross‐sections from any direction. FIB‐tomography has been previously described for a few biological samples,^[^ ^8^, ^34^ ^]^ but the analysis was limited to a qualitative space group assignment. Perhaps constrained by the reconstruction process, no in depth structural characterization was performed on the reconstructed volumes. The main limiting factor is not the inherent instrument resolution, but lies in the sample preparation. A backfilling step of the air‐cuticle material is necessary for any 3D reconstruction resulting from a slice‐and‐view process to limit the depth of field and to guarantee a sufficient contrast for accurate automated binarization. This is usually achieved through filling of the sample with a low viscosity resin prior to tomography,^[^ ^39^ ^]^ a time‐consuming ex situ process that is often ill‐controlled, and ultimately yields a poor image contrast.

As part of this study, we developed an imaging approach based on in situ CVD‐based filling inspired by Eswara‐Moorty et al.^[^ ^40^ ^]^ Platinum (Pt) was deposited into the porous structure using an electron‐beam induced deposition process (EBID). A precursor gas containing Pt was injected close to the region of interest (ROI) and decomposed into the air phase by interacting with a secondary electron beam (for details, see Experimental Section). Choosing Pt backfilling was primarily motivated by the ease of the in situ process, allowing precise control over the area and depth of deposition in the electron microscope. This backfilling tremendously benefited image acquisition and analysis due to the enhanced contrast between the Pt and the chitin network, as well as reducing curtaining effects and charge accumulation (see Figure S1, Supporting Information). Another advantage was the preservation of the integrity of the structure by protection against beam‐induced melting and deformation. The combination of all these parameters enables a great fidelity of the reconstruction and better reliability of the structural analysis compared to other methods.

### 2.4 3D Volumetric Imaging of Weevil Scales

**Figure 3:**
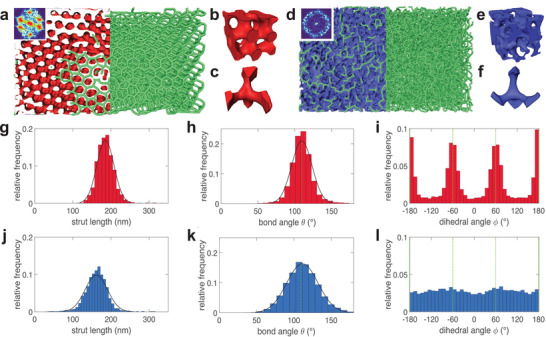
a,d) Full 3D reconstruction (left) and skeletonization (right) of the chitinous structures present in the red and blue scales, *V* = 5 × 4 × 3 µm^3^. Both structures are seen from the top, that is, the plane corresponds to *x*–*z*. Insets: cross‐section of the respective 3D FFTs. b,e) Small volume *V* ≈ (550 nm)^3^ and (c,f) single node reconstruction. (g,j) Strut length distributions for similar strut counts (*n*_red_ = 5208, *n*_blue_ = 3494). h,k) Bond angle distributions (*n*_red_ = 18571, *n*_blue_ = 21139). i,l) Dihedral angle distributions (*n*_red_ = 28568, *n*_blue_ = 22014). (a–c, g–i): red scale, (d–f, j–l): blue scale.

Using this backfilling procedure, FIB‐tomography was performed using a slice‐and‐view process and the obtained image stacks were converted into 3D reconstructions (see Experimental Section for details). Note that since the cortex was removed prior to the tomography, only the structure within the scale was imaged. In brief, the main workflow to reconstruct 3D structures consisted of a registration step, followed by cropping, filtering, and segmentation for reconstruction. The reconstruction resulted in two data‐sets of 5 × 4 × 3 µm^3^ with a voxel resolution of 10 nm edge length. This enabled the visualization and structural analysis of sizable volumes (**Figure** **3**a,d), the extraction of supercells (Figure 3b,e) and single repeat units (Figure 3c,f). Additional visuals are shown in Figure S4a–d, Supporting Information. The chitin filling fraction estimated from the binary voxelized volume was 42% for the red scale, matching the estimation from the 2D area fraction, and 32% for the blue scale. Note that a precise determination of filling fraction is challenging as it is highly dependent on the threshold and the algorithm used during the binarization process. The values obtained here are in the range of previously reported values for related *Pachyrhynchus* ^[^ ^9^, ^30^ ^]^ and *Entimus* ^[^ ^35^ ^]^ weevils, and are not far from the optimal filling fraction that maximizes the frequency width of the optical bandgap of a single‐diamond PC.^[^ ^35^ ^]^

The full reconstructed volume of the red scale comprised multiple domains with recognizable patterns (Figure S4a,b, Supporting Information), although the top plane remained in the (111) orientation throughout. The unit cell appeared as a well‐defined structure made of regularly connected tetrahedral units (Figure 3b,c). The unit cell was identical throughout the entire volume, as long as it was extracted away from a grain boundary.

The structure within the blue scale exhibited no visible grain boundaries and the unit cell appeared irregular and tortuous (Figure 3d,e and Figure S4c,d, Supporting Information). Tetrahedral arrangements of nodes were also observed (Figure 3f) but the connectivity was less uniform compared to the ordered structure of the red scales. In particular, a number of trigonal planar nodes arrangements were present, decreasing the average coordination number. Cross‐sectioning the 3D FFT of each structure yielded similar patterns to the one observed in 2D, that is, a hexagonal pattern for the ordered structure and a well‐defined ring for the amorphous one (insets in Figure 3a,d), indicative of a full 3D isotropy.

To fully characterize the diamond structure of the red scales and to investigate a possible underlying order in the blue scales, we performed a skeletonization step followed by a morphological study. Based on the skeleton of each structure, the average coordination number of each vertex (or node) was extracted as well as the distributions of the strut lengths, bond angles between connected segments and dihedral (or torsion) angles between intersecting planes (Figure 3g–l). The reconstructed red scale possessed an average strut length *L*_r_ = 185 ± 22 nm (*n* = 5208), an average coordination number *CN*_r_ = 3.8 ± 0.4 (*n* = 3949) and an average bond angle θ_r_ = 109.6 ± 13.9°  (*n* = 18571). The same study conducted on a blue scale over a comparable subvolume yielded *L*_b_ = 162 ± 29 nm (*n* = 3494), *CN*_b_ = 3.4 ± 0.6 (*n* = 9983), θ_b_ = 111.3 ± 21.0°  (*n* = 21139).

In both cases, the values of strut lengths and bond angles followed a Gaussian distribution, with the disordered network having a slightly higher standard deviation. The statistical analysis of the ordered network yielded values of node connectivity and bond angles close to the values expected for the ideal diamond, that is, *CN*_d_ = 4 and θ_d_ = 109.5°. The disordered network showed a slightly lower average connectivity but similar average bond angles. Note that the angle distribution is a more robust parameter than the connectivity as it is less prone to reconstruction artifacts. Because of binarization and skeletonization artifacts, there is a tendency to underestimate the coordination number and since the average bond angle is close to the ideal tetrahedral angle, it seems that the node positions of the disordered network are consistent with a tetrahedral distribution. The dihedral angle distribution of both scales showed local maxima around the values expected for a diamond, that is, ϕ_d_ ∈ { ± 180; ± 60}°, but while the ordered network yielded well‐defined, narrow peaks, the disordered network shows a nearly constant angular distribution with only a minor undulation. The blue scale thus appears much more tortuous than the red scale at medium range (Figure 3b,e), which is a result of about random torsion angles between tetrahedral units.

Some part of the distribution width of the measured quantities can be attributed to the data acquisition and reconstruction processes, combined with topological and positional defects accounting for grain boundaries and other inherent imperfections, and defects from the biological processes that generated the network structures.^[^ ^28^, ^41^, ^42^ ^]^ Since these contributions should be identical for both systems, the wider spread observed for all measured quantities in the blue scale can be ascribed to a higher “disorder”. The difference is most notable in the dihedral angle distribution, which is coherent with decreasing structural correlations on medium to long length scales.

**Figure 4:**
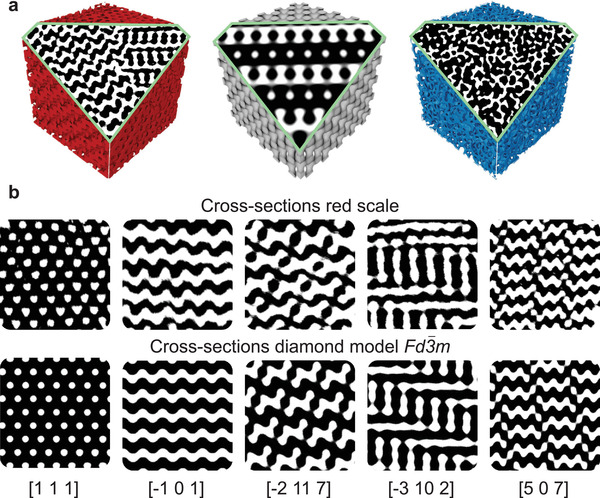
a) Illustration of how cross‐sections are extracted from reconstructed volumes: red scale, diamond model, blue scale. The border of the cross‐section is shown in green, the pattern shows chitin in white and air in black. b) Matching cross‐sections between the tomogram of the red scale (top row) and the model diamond (bottom row). Chitin is shown in white, air in black.

The sizable reconstructed volume also allows extracting TEM‐like cross‐sections that can be compared to cross‐sections of known space groups generated by level‐set equations. A qualitative analysis of these cross‐sections can already shed light on the nature of the networks and reveal any anisotropy. This method can be used in combination with methods to attribute unknown structures to a space group.^[^ ^35^, ^43^ ^]^ To further confirm the $Fd\bar{3}m$ space group of the red structure, planes exhibiting a recognizable pattern were extracted from the volume and compared to the ideal diamond lattice generated from the level‐set equation. To this end, the computed model was rotated and cross‐sectioned incrementally until planes matching the experimental data were found. **Figure** **4** shows compelling resemblances between the cross‐sections of the red scales and the single‐diamond model, confirming the space group $Fd\bar{3}m$ of the red scale. Other 3D network geometries were similarly investigated, such as the single‐network gyroid structure (space group *I*4_1_32), which is also often found in insects,^[^ ^10^, ^29^ ^]^ but no matching cross‐sections were consistently found.

The same procedure was carried out for the reconstructed internal structure of the blue scale and yielded similar‐looking cross‐sections regardless of orientation, which is consistent with the apparent isotropy of the network. Observations made along the main directions ([001], [100], [010], [110], [111]) are shown in Figure S4e, Supporting Information. Since no recognizable pattern appeared, this scale obviously does not possess a well‐ordered 3D symmetric structure.

### 2.5 Volumetric Structural Order

**Figure 5:**
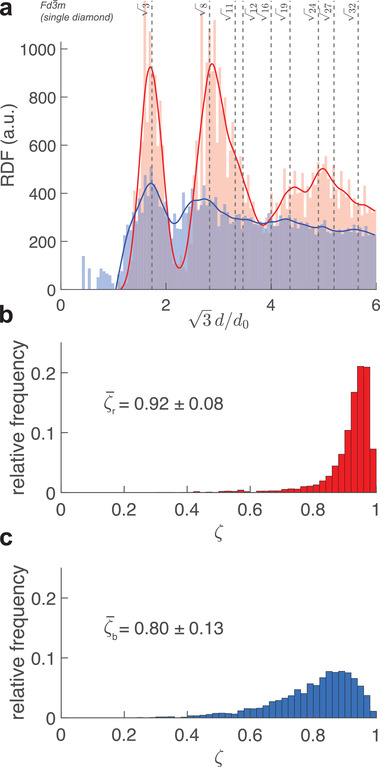
a) Radial distribution function computed for two scales. The abscissa is normalized by $d_{0}/\sqrt{3}$ where *d*_0_ is the average strut length. Histogram distributions of the scales are shown in translucent red and blue to match the color of the scales they correspond to. Cubic splines are shown as opaque lines of the same color. The peak positions of the single diamond $Fd\bar{3}m$ are indicated as vertical dashed lines. b) Distribution of the tetrahedral order parameter ζ calculated from a subvolume of the red scale (*n*_nodes_ = 2406) and c) the blue scale (*n*_nodes_ = 3161).

The main distinction between an ordered and a quasi‐ordered structure is that the latter only possesses short range order, that is, local order of the scattering centers gives way to disorder on longer length scales. Radial distribution functions (RDF) of the vertex positions in each sample^[^ ^44^ ^]^ were computed to estimate the distance over which local order disappears. **Figure** **5**a shows the calculated RDF for the two obtained volumes.

The red sample displays clear local peaks expected for a diamond structure with maxima at $\sqrt{3},\sqrt{8},\sqrt{19}$, and $\sqrt{24}$ of the scaled distance *d*/*d*_0_, calculated from the reflection conditions of the $Fd\bar{3}m$ space group.^[^ ^45^ ^]^ The RDF of the blue scales, however, shows a broad background and peaks that are much less pronounced compared to those of the red scale, characteristic of a broad distribution of nearest neighbor distances. Nevertheless, the blue scale clearly displays the first two $Fd\bar{3}m$ peaks, revealing short‐range order and a structural similarity to a diamond network. The absence of higher‐order peaks attests the lack of long‐range order, similar to amorphous solids.^[^ ^44^, ^46^ ^]^

To further quantify the degree of short‐range order on the scale of single repeat units, a tetrahedral order parameter was calculated for every node with a fourfold connectivity (Figure 5b,c). For this, we used a rescaled version of the order parameter introduced by Chau et al.^[^ ^47^ ^]^ via

(1) $$\zeta =1-\frac{3}{8}\sum_{j=1}^{3}\sum_{k=j+1}^{4}{\left(\cos\;\theta_{jk}+\frac{1}{3}\right)}^{2}$$

where θ_*jk*_ is the bond angle between each edge connected to the same node. In a four‐connected structure with randomly oriented edges, ${\bar{\zeta }}_{\mathrm{rand}}\;=0$. In a perfectly periodic diamond network, all bond angles are $\cos^{-1}\left(\theta_{jk}\right)=-\frac{1}{3}$ such that ${\bar{\zeta }}_{d}=\zeta_{d}=1$ for all *j*, *k* values. An analysis of the reconstructed scales yielded average tetrahedral order parameters of ${\bar{\zeta }}_{r}=0.92\pm 0.08$ for the red scale and ${\bar{\zeta }}_{b}=0.80\pm 0.13$ for the blue scale, which is consistent with their respective bond angle distributions. The shape of the distributions, with only one local maximum centered around a value close to 1, indicates that both networks have quite uniform local topologies across the entire investigated volumes similar to that of an ideal diamond lattice.^[^ ^48^ ^]^

### 2.6 Optical Modeling and Bandgap Criteria

**Figure 6:**
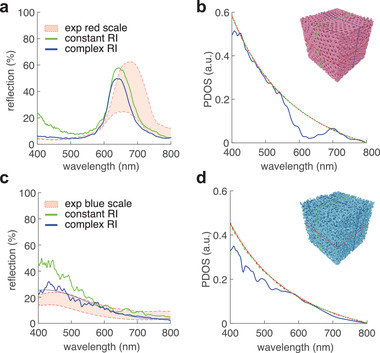
Simulated reflection spectra based on the 3D tomogram a) of a red scale and c) of a blue scale with the refractive index dispersion of unpigmented chitin (green line) and pigmented chitin (blue line). The experimental spectra are shown as red shaded areas. Photonic density of states for the b) red and d) blue scales were calculated for the unpigmented chitin, plotted in blue. The theoretical and simulated PDOS for an equivalent homogeneous medium are plotted in red and green, respectively. Insets: 3D volumes used as input for the FDTD simulations.

To understand the optical function of both networks and validate our reconstruction process, FDTD calculations based on the acquired volumetric data were performed. Simulations resulted in the reflection spectra shown in **Figure** **6**a,c, which were compared with experimental data (red‐shaded bands show the experimental variations).

In a first approximation, the refractive index of chitin was set to *n*_c_ = 1.56 for the entire spectrum (green lines in Figure 6a,c). These simulation results deviated significantly from the experimental data at wavelengths below 500 nm. The assumption of a wavelength‐dependent dispersion of the complex refractive index, accounting for both the wavelength dependence of the chitin^[^ ^6^ ^]^ as well as the absorption coefficient of the pigment within the chitin (for details, see Experimental Section), produced simulated spectra that improved the match with the experimental ones, both in terms of peak wavelength and overall reflection intensity particularly at short wavelengths where pigment absorption becomes significant (blue lines in Figure 6a,c). The blue shift of the simulated spectra compared to the directly measured data may arise from a different scale location or from a slight compression of the scales during processing of the volumetric data.

A further important aspect is the presence of a pigment in both types of scales. The role of the broad‐band absorbing pigment is primarily to suppress short wavelength reflections and reduce multiple scattering—as previously observed in other animals and manufactured systems.^[^ ^49^, ^50^, ^51^ ^]^ In the case of the red scales, the pigment is crucial in suppressing incoherently scattered blue light from the defects in the scale structure. Without the pigment, the scale would have an unsaturated, more purplish appearance (Figure 6a, green line). Blue structural coloration is possible without the pigment contribution (Figure 6c, green line), but also here, the pigment suppresses scattered stray light and stabilizes the hue. Overall, the good match of the simulated and measured reflection spectra in Figure 6a,c, using the reconstructed scales as an input, validates our tomography and reconstruction protocol, which should be readily expandable to other porous media with similar internal length scales.

To determine the physical origin of this coloration, the photonic densities of states (PDOS) were calculated for each scale, shown in Figure 6b,d (blue lines). In this calculation, a constant refractive index of 1.56 was used, that is, only the structural contribution was taken into account. These were compared to the theoretical and simulated PDOS of equivalent homogeneous media (Figure 6b,d, red and green lines, respectively). In these equivalent media, the RI was taken as a filling fraction‐weighted average of the refractive indices of chitin and air. For both cases, the PDOS determined from the scale networks show significant deviations from the expected quadratic behavior.^[^ ^52^ ^]^ The ordered structure displays a clear dip between 545–695 nm, which corresponds to the position of the simulated reflection peak. The dip almost reaches zero and possesses a gap width to mid‐gap frequency ratio of 24%. The deviation of the disordered structure is more subtle as no localized dip is observed in the simulated range. Nevertheless, at low wavelengths, the PDOS values are significantly lowered with respect to the homogeneous medium, giving rise to a broad partial bandgap ranging from 400 to 595 nm, in accordance with the simulated reflection peak of the corresponding structure.

Although a bandgap is expected for a “perfect” diamond, the partial bandgap of the ordered structure shows a high resilience of the optical properties with respect to structural imperfections, such as grain boundaries, imperfect periodicity, positional and topological defects.^[^ ^41^ ^]^ In an amorphous medium, the incoming light undergoes strong isotropic scattering usually resulting in white coloration. The presence of a bandgap requires a sufficient RI contrast between the two phases, structural correlations on at least a local scale to enable a wavelength‐selective reflection process through coherent scattering,^[^ ^21^, ^53^ ^]^ and minimization of long‐range fluctuations to inhibit diffuse scattering.^[^ ^54^ ^]^ The investigation of amorphous 3D network structures in biological systems leading to a partial optical bandgap has recently gained increased traction. For instance, investigated structures resulting from spinodal decomposition or random close packing of spheres are known to generate colors (often blue) in bird feathers and beetle scales.^[^ ^25^, ^26^, ^27^ ^]^ Recently, model calculations by Edagawa and colleagues introduced an amorphous network exhibiting a complete bandgap, the so‐called photonic amorphous diamond (PAD), a tetrahedral network with only short‐range order, as opposed to the photonic crystalline diamond (PCD) which also possesses long‐range and translational order.^[^ ^32^ ^]^ Surprisingly, the PAD bandgap was of almost the same quality as the PCD. Sellers et al. showed that the formation of a bandgap is favored by connected networks displaying low (< 5) and uniform coordination numbers. Amorphous structures with trigonal and tetragonal strut connections (connectivities of three and four, respectively) are champion structures for isotropic partial bandgaps, due to the high symmetry of their scattering centers.^[^ ^28^, ^55^, ^56^ ^]^

The criterion for symmetry is the ability to superimpose scattering centers upon rotation and permutation, which requires, in addition to a uniform connectivity, a narrow distribution of bond angles. Our results corroborate this hypothesis by comparing the networks of the two scale types, which, first, possess a relatively uniform connectivity that lies between three and four, and, second, possess scattering centers with high symmetry, with values of the tetrahedral order parameter reaching 0.80 and 0.92. Additionally, the less periodic structure of the blue scale, which displays a wider spread in coordination number and bond angles, exhibits a PBG which is less localized and intense compared to the periodic diamond morphology of the red scale (Figure 6b,d).

## 3 Conclusion

The investigated specimen is set apart from other *Pachyrhynchys* weevils by its display of two separate sets of colored scales, each comprising a structure with a different degree of order. While the coexistence of both ordered and disordered inner structures on a single specimen has previously been reported in the scales of other beetles (e.g., *Eupholus magnificus*,^[^ ^23^ ^]^ *Sulawesiella rafaelae*,^[^ ^24^ ^]^ and *Sternotomis pulchra bifasciata* ^[^ ^29^ ^]^) these studies have either mostly focused on their optical properties with structural information being inferred from 2D cross‐sections only or were limited in their characterization to structure diagnosis. An in‐depth characterization of the network structures is, however, critical not only to understand the optical properties, but also the underlying cellular processes leading to their formation, which could inform new approaches to the design and manufacture of bio‐inspired optical devices with different degrees of order.^[^ ^57^, ^58^ ^]^ Although the precise intracellular mechanism of scale formation in beetles is unknown, it is possibly homologous to the growth of butterfly scales that form as a result of the co‐assembly of an infolding lipid‐bilayer membrane and cuticle in the lumen of the developing scale.^[^ ^10^, ^59^, ^60^ ^]^

The present work allows the first direct comparison of structural coloration arising from a crystalline structure and its amorphous counterpart, supported by optical investigations, 3D structural data analysis as well as simulations. The 3D analyses show that structural differences lie at the heart of the difference in spectral and spatial dependency between the red and blue scales. Local order was observed in both structures, with a fixed strut length and a narrow distribution of angles leading to a high value of their respective tetrahedral order parameter. However, the circular pattern of the FFT, the shape of the RDF and the wide distribution of dihedral angles for the blue scale clearly show a lack of long‐range order compared to the ordered red scale, and is characteristic of the quasi‐ordered nature of the network. Diamond structures are known to open an optical bandgap both in their crystalline (PCD) and amorphous form (PAD),^[^ ^32^ ^]^ yet most studies on PADs have been purely computational and yielded substantial bandgaps only for high dielectric contrast (Si‐air, i.e., 13:1). Our results show an experimental sample displaying both a PCD and a PAD capable of generating color at low dielectric contrasts (chitin‐air, i.e., 2.4:1). An optical bandgap, even if only partial, in a low dielectric contrast quasi‐ordered material is remarkable and carries significance for the design and fabrication of quasi‐ordered PC.

## 4 Experimental Section

### Specimen

*P. congestus mirabilis* weevils (Billberg 1820; Curculionidae: Entiminae: Pachyrhynchini) native to the Philippines were purchased from thebugmaniac.com.

### Optical Characterization

Spectral characterization was performed using a xenon light source (Thorlabs SLS401; Thorlabs GmbH, Dachau, Germany) and a ZEISS Axio Scope.A1 microscope (Zeiss AG, Oberkochen, Germany). The light reflected from the sample was collected by an optical fiber (230 µm core) in a plane confocal to the image plane, resulting in an effective measurement spot diameter of ≈13 µm at a magnification of × 20. The spectra were analyzed by a spectrometer (Ocean Optics Maya2000 Pro; Ocean Optics, Dunedin, FL, USA), a white Lambertian diffuser served as a reference. Optical micrographs were captured with a CCD camera (GS3‐U3‐28S5C‐C, FLIR Integrated Imaging Solutions Inc., Richmond, Canada). Reflection spectra were taken on scales still attached to the elytron. Transmission spectra were also taken with the same equipment, but the scales were first detached from the elytron and deposited on a microscope glass slide using a scalpel, before immersion in a refractive index matching oil (*n*_o_ = 1.55). Scatterometry measurements were performed by placing a Bertrand lens (Zeiss 453671) into the imaging pathway and using a high numerical aperture air objective (Zeiss Epiplan Neofluar 100×, NA 0.9). This allowed measurements of scattering angles up to 64°.

### Electron Microscopy and Tomography

Individual scales were gently scratched from the elytron and deposited on an aluminum SEM stub (Plano‐EM, Wetzlar, Germany) covered with conductive carbon tape. The cortex layer surrounding individual scales was partially removed by plasma etching using a PE‐100‐RIE system (Plasma Etch Inc., Nevada, USA). Samples were exposed to a 4:20 O_2_/Ar plasma mixture for 12 to 18 min. The stub was then sputter coated with a 7 nm thick layer of either gold or platinum using a Cressington 208 HR (Cressington Scientific Instruments, Watford, England). Copper tape and silver paste were also added to increase the conductivity of the sample and limit charging effects. Top‐view SEM pictures were taken using a Tescan Mira3 (Tescan, Brno, Czechia) with a beam voltage of 8 kV and a working distance of 10 mm. Cross‐sections were milled and imaged using a Thermo Scientific Scios 2 DualBeam FIB‐SEM (FEI, Eindhoven, the Netherlands).

To enable 3D reconstruction, the scales were first filled in situ with platinum using an electron beam‐induced deposition (Pt‐EBID) process inspired by Eswara‐Moorthy et al.^[^ ^40^ ^]^ A gaseous precursor, C_9_H_16_Pt, was injected near the surface of the cortex‐free ROI through the gas‐injection system needle, and dissociated into Pt by interacting with the electron beam set to an acceleration voltage of 30 kV, a current of 1.6 nA and a dwell time of 15 µs. These parameters were optimized to enable a complete infiltration throughout the entire scale thickness. A rough cut was milled in front of the ROI to expose the imaging plane and trenches were milled on the sides to provide a deposition site for the milled material and prevent redeposition on the exposed section. A fiducial was created to limit beam‐ and tilt‐ induced drift, as well as stage displacement. The tomography process was automatized using ThermoFisher Auto Slice and View software (v. 4). Slices of 10 to 30 nm thickness were milled with the Ga^+^ beam set at 30 kV acceleration voltage and 0.30 nA beam current. Images were acquired after each slice in the OptiTilt configuration using the built‐in SEM Everhart‐Thornley (ETD, secondary electrons) and in‐lens T1 (A+B composite mode, back‐scattered electrons) detectors set to a voltage of 2 kV. The image distortion induced by the acquisition at a 52° angle was compensated with the built‐in tilt correction feature.

### Reconstruction

Stacks of images were processed using Fiji^[^ ^61^ ^]^ and FEI Avizo for Materials Science 2020.2 software for basic image processing, 3D reconstruction, and statistical analysis. Registration was performed using a combination of the Fiji *StackReg* ^[^ ^62^ ^]^ and *Correct 3D Drift* plug‐ins.^[^ ^63^ ^]^ A median filter was applied to despeckle and smooth the images, which were then inverted to make the chitinous material appear white and the Pt filling (corresponding to the air network) black. Segmentation was done using the trainable Weka segmentation 3D plug‐in^[^ ^64^ ^]^ in Fiji. The images were sampled down to yield isotropic voxels and to limit artifacts. Skeletonization was then performed in Avizo using an algorithm based on distance mapping and thinning. The study of the structure revealed anisotropy in the direction normal to the scale which was found in all scales of the *Pachyrhynchus* family and systematically led to a bimodal strut length distribution. As previous small‐angle X‐ray scattering studies did not reveal this anisotropy,^[^ ^29^, ^30^ ^]^ this is believed to arise from the FIB‐SEM imaging process. The images were thus corrected by applying a vertical stretch of ≈1.2. The implementation of this step lead to isotropic segment lengths, in line with previously reported data. The strut length distribution, average coordination number (CN) and filling fraction were computed using Avizo built‐in features. The strut lengths were only computed on struts connected to nodes with fourfold connectivity to limit artifacts due to struts with uneven radii that were disconnected by the skeletonization algorithm. Bond and dihedral angles were computed in Matlab v.2019b from the positions of all connected nodes derived from the skeleton (see Figure S2, Supporting Information).

### Optical Modeling

The optical properties of an idealized single network diamond (space group $Fd\bar{3}m$), approximated by Schwarz's D triply periodic minimal surface model, was generated from its level‐set equation following the IMDS (inter‐material dividing space) method,^[^ ^43^, ^65^ ^]^ cos (2π*z*/*a*) sin (2π(*x* + *y*)/*a*) + sin (2π*z*/*a*) cos (2π(*x* − *y*)/*a*) = *t*, where *a* is the cubic lattice constant and *t* is the level‐set parameter that determines the chitin filling fraction *v*_f_ by the relation *t* = 2.4 (*v*_f_ − 0.5). The models were generated using Matlab with a unit cell *a* chosen to match the lattice parameter determined from the SEM images.

FDTD calculations that directly used the obtained 3D reconstructions as input were performed using the Lumerical software (Lumerical FDTD Solutions, v. 8.20; Ansys Inc, Canonsburg, PA, USA) to predict reflection spectra and photonic density of states (PDOS). The refractive index of the material was chosen to be a complex function of the wavelength to take into account both the effects of the chitin and the absorbing pigment present in the scales. Since the pigment contribution was mainly absorptive, the real part was taken equal to the chitin RI which was well approximated by a wavelength‐dependent Cauchy law (see Equation (S1), Supporting Information).^[^ ^33^ ^]^ The imaginary part of the RI (the absorption coefficient) was calculated from the experimental transmission spectra taken in a refractive index matching fluid (see SI, Equation (S2) and Figure S3, Supporting Information).

For local PDOS calculations, three dipoles, each oriented along one direction (*x*, *y*, *z*), were placed in proximity of the structure and the electrical field inside the structure was calculated using a transmission box surrounding it. The total local density of states was subsequently calculated based on the imaginary part of Green's function.^[^ ^52^ ^]^

### Statistical Analysis

A single *P. congestus mirabilis* specimen was investigated. The long and short diameters of blue and red scales were measured using Fiji.^[^ ^61^ ^]^ The optical spectra showed one representative curve for each patch of color observed on the elytron. The tomography datasets and subsequent statistical analysis were carried out on one blue scale and one red scale. The data were processed as described in Experimental Section. All values were presented as means and standard deviations (mean ± SD). The statistical analysis and data processing were carried out using Matlab R2019b (Mathworks).

## Conflict of Interest

The authors declare no conflict of interest.

## Author Contributions

K.D., U.S., and B.D.W. contributed to the conceptualization; K.D. contributed to the investigation of optical measurements, SEM imaging, FIB‐tomography and reconstruction, data curation, visualization, and wrote the original draft; K.D. and B.D.W. contributed to the optical simulations; B.D.W. and U.S. contributed to the supervision; U.S. and B.D.W. contributed to the funding acquisition; all authors contributed to writing and editing.

## Supporting information

Supporting Information

## Data Availability

The data that support the findings of this study are available at the Zenodo data repository (https://doi.org/10.5281/zenodo.6783691).

## References

[1] I. C. Cuthill , W. L. Allen , K. Arbuckle , B. Caspers , G. Chaplin , M. E. Hauber , G. E. Hill , N. G. Jablonski , C. D. Jiggins , A. Kelber , J. Mappes , J. Marshall , R. Merrill , D. Osorio , R. Prum , N. W. Roberts , A. Roulin , H. M. Rowland , T. N. Sherratt , J. Skelhorn , M. P. Speed , M. Stevens , M. C. Stoddard , D. Stuart‐Fox , L. Talas , E. Tibbetts , T. Caro , Science 2017, 357, eaan0221. 28774901 10.1126/science.aan0221

[2] C. Y. Lee , S. P. Yo , R. W. Clark , J. Y. Hsu , C. P. Liao , H. Y. Tseng , W. S. Huang , J. Zool. 2018, 306, 36.

[3] S. Vignolini , M. P. Davey , R. M. Bateman , P. J. Rudall , E. Moyroud , J. Tratt , S. Malmgren , U. Steiner , B. J. Glover , New Phytol. 2012, 196, 1038. 23043621 10.1111/j.1469-8137.2012.04356.x

[4] J. Tinbergen , B. D. Wilts , D. G. Stavenga , J. Exp. Biol. 2013, 216, 4358. 24031051 10.1242/jeb.091561

[5] L. D'Alba , L. Kieffer , M. D. Shawkey , J. Exp. Biol. 2012, 215, 1272. 22442364 10.1242/jeb.064907

[6] D. G. Stavenga , Mater. Today Proc. 2014, 1, 109.

[7] H. M. Whitney , M. Kolle , Science 2009, 323, 130. 19119235 10.1126/science.1166256

[8] J. W. Galusha , L. R. Richey , J. S. Gardner , J. N. Cha , M. H. Bartl , Phys. Rev. E 2008, 77, 050904. 10.1103/PhysRevE.77.050904 18643018

[9] V. Welch , V. Lousse , O. Deparis , A. Parker , J. P. Vigneron , Phys. Rev. E 2007, 75, 041919‐1. 10.1103/PhysRevE.75.041919 17500933

[10] B. D. Wilts , B. A. Zubiri , M. A. Klatt , B. Butz , M. G. Fischer , S. T. Kelly , E. Spiecker , U. Steiner , G. E. Schröder‐Turk , Sci. Adv. 2017, 3, e1603119. 28508050 10.1126/sciadv.1603119 PMC5406134

[11] E. Yablonovitch , Phys. Rev. Lett. 1987, 58, 2059. 10034639 10.1103/PhysRevLett.58.2059

[12] L. Martin‐Moreno , F. Garcia‐Vidal , A. Somoza , Phys. Rev. Lett. 1999, 83, 73.

[13] J. D. Joannopoulos , S. Johnsons , J. Winn , R. Meade , Photonic Crystals: Molding the Flow of Light, Princeton University Press, Princeton 2008.

[14] C. F. Bohren , Am. J. Phys. 1987, 55, 524.

[15] B. D. Wilts , X. Sheng , M. Holler , A. Diaz , M. Guizar‐Sicairos , J. Raabe , R. Hoppe , S. H. Liu , R. Langford , O. D. Onelli , D. Chen , S. Torquato , U. Steiner , C. G. Schroer , S. Vignolini , A. Sepe , Adv. Mater. 2018, 30, 1702057. 10.1002/adma.201702057 28640543

[16] P. Vukusic , B. Hallam , J. Noyes , Science 2007, 315, 348. 17234940 10.1126/science.1134666

[17] S. L. Burg , A. Washington , D. M. Coles , A. Bianco , D. McLoughlin , O. O. Mykhaylyk , J. Villanova , A. J. Dennison , C. J. Hill , P. Vukusic , S. Doak , S. J. Martin , M. Hutchings , S. R. Parnell , C. Vasilev , N. Clarke , A. J. Ryan , W. Furnass , M. Croucher , R. M. Dalgliesh , S. Prevost , R. Dattani , A. Parker , R. A. Jones , J. P. A. Fairclough , A. J. Parnell , Commun. Chem. 2019, 2, 100.

[18] G. Barkema , N. Mousseau , Phys. Rev. B 2000, 62, 4985.

[19] C. E. Zachary , S. Torquato , J. Stat. Mech.: Theory Exp. 2009, 2009, P12015.

[20] K. Edagawa , Sci. Technol. Adv. Mater. 2014, 15, 034805. 27877676 10.1088/1468-6996/15/3/034805 PMC5090521

[21] H. Yin , B. Dong , X. Liu , T. Zhan , L. Shi , J. Zi , E. Yablonovitch , Proc. Natl. Acad. Sci. U. S. A. 2012, 109, 10798. 22615350 10.1073/pnas.1204383109 PMC3390825

[22] B. Q. Dong , T. R. Zhan , X. H. Liu , L. P. Jiang , F. Liu , X. H. Hu , J. Zi , Phys. Rev. E 2011, 84, 011915. 10.1103/PhysRevE.84.011915 21867221

[23] C. Pouya , D. G. Stavenga , P. Vukusic , Opt. Express 2011, 19, 11355. 21716365 10.1364/OE.19.011355

[24] E. Bermúdez‐Ureña , C. Kilchoer , N. P. Lord , U. Steiner , B. D. Wilts , iScience 2020, 23, 101339. 32688285 10.1016/j.isci.2020.101339 PMC7371903

[25] L. Shi , Y. Zhang , B. Dong , T. Zhan , X. Liu , J. Zi , Adv. Mater. 2013, 25, 5314. 24089349 10.1002/adma.201301909

[26] V. Saranathan , J. D. Forster , H. Noh , S. F. Liew , S. G. Mochrie , H. Cao , E. R. Dufresne , R. O. Prum , J. R. Soc. Interface 2012, 9, 2563. 22572026 10.1098/rsif.2012.0191 PMC3427513

[27] M. D. Shawkey , V. Saranathan , H. Pálsdóttir , J. Crum , M. H. Ellisman , M. Auer , R. O. Prum , J. R. Soc. Interface 2009, 6, S213. 10.1098/rsif.2008.0374.focus PMC2706473 19158016

[28] S. R. Sellers , W. Man , S. Sahba , M. Florescu , Nat. Commun. 2017, 8, 14439. 28211466 10.1038/ncomms14439 PMC5321726

[29] V. Saranathan , A. E. Seago , A. Sandy , S. Narayanan , S. G. Mochrie , E. R. Dufresne , H. Cao , C. O. Osuji , R. O. Prum , Nano Lett. 2015, 15, 3735. 25938382 10.1021/acs.nanolett.5b00201

[30] B. D. Wilts , V. Saranathan , Small 2018, 14, 1802328. 10.1002/smll.201802328 30112799

[31] Y. Chang , Y. Ogawa , G. Jacucci , O. D. Onelli , H.‐y. Tseng , S. Vignolini , Adv. Opt. Mater. 2020, 8, 2000432.

[32] K. Edagawa , S. Kanoko , M. Notomi , Phys. Rev. Lett. 2008, 100, 013901. 18232763 10.1103/PhysRevLett.100.013901

[33] D. G. Stavenga , H. L. Leertouwer , T. Hariyama , H. A. De Raedt , B. D. Wilts , PLoS ONE 2012, 7, e49743. 23185423 10.1371/journal.pone.0049743 PMC3502265

[34] R. Ebihara , H. Hashimoto , J. Kano , T. Fujii , S. Yoshioka , J. R. Soc. Interface 2018, 15, 145. 10.1098/rsif.2018.0360 PMC6127177 30089688

[35] B. D. Wilts , K. Michielsen , J. Kuipers , H. De Raedt , D. G. Stavenga , Proc. R. Soc. B 2012, 279, 2524. 10.1098/rspb.2011.2651 PMC3350696 22378806

[36] P. Vukusic , D. G. Stavenga , J. R. Soc. Interface 2009, 6, S133. 19158009 10.1098/rsif.2008.0386.focus PMC2706471

[37] C. Kizilyaprak , D. D. Bellis , W. Blanchard , J. Daraspe , B. M. Humbel , Biol. Field Emiss. Scan. Electron Microsc. 2019, 2, 545.

[38] V. Saranathan , C. O. Osuji , S. G. Mochrie , H. Noh , S. Narayanan , A. Sandy , E. R. Dufresne , R. O. Prum , Proc. Natl. Acad. Sci. U. S. A. 2010, 107, 11676. 20547870 10.1073/pnas.0909616107 PMC2900708

[39] J. W. Galusha , L. R. Richey , M. R. Jorgensen , J. S. Gardner , M. H. Bartl , J. Mater. Chem. 2010, 20, 1277.

[40] S. K. Eswara‐Moorthy , P. Balasubramanian , W. Van Mierlo , J. Bernhard , M. Marinaro , M. Wohlfahrt‐Mehrens , L. Jörissen , U. Kaiser , Microsc. Microanal. 2014, 20, 1576. 25088604 10.1017/S1431927614012884

[41] S. R. Mouchet , S. Luke , L. T. McDonald , P. Vukusic , Faraday Discuss. 2020, 223, 9. 33000817 10.1039/d0fd00101e

[42] L. S. Tsimring , Rep. Prog. Phys. 2014, 77, 026601. 24444693 10.1088/0034-4885/77/2/026601 PMC4033672

[43] K. Michielsen , D. G. Stavenga , J. R. Soc. Interface 2008, 5, 85. 17567555 10.1098/rsif.2007.1065 PMC2709202

[44] R. Zallen , The Physics of Amorphous Solids, Wiley, New York 1998.

[45] T. Hahn , International Tables for Crystallography, vol. A, Springer, Cham 2005, p. 911.

[46] C. Jin , X. Meng , B. Cheng , Z. Li , D. Zhang , Phys. Rev. B 2001, 63, 195107.

[47] P.‐L. Chau , A. Hardwick , Mol. Phys. 1998, 93, 511.

[48] S. F. Liew , J. Forster , H. Noh , C. F. Schreck , V. Saranathan , X. Lu , L. Yang , R. O. Prum , C. S. O'Hern , E. R. Dufresne , H. Cao , Opt. Express 2011, 19, 8208. 21643071 10.1364/OE.19.008208

[49] J. D. Forster , H. Noh , S. F. Liew , V. Saranathan , C. F. Schreck , L. Yang , J. C. Park , R. O. Prum , S. G. Mochrie , C. S. O'Hern , H. Cao , E. R. Dufresne , Adv. Mater. 2010, 22, 2939. 20414884 10.1002/adma.200903693

[50] G. Jacucci , S. Vignolini , L. Schertel , Proc. Natl. Acad. Sci. U. S. A. 2020, 117, 23345. 32900921 10.1073/pnas.2010486117 PMC7519302

[51] Y. Hu , D. Yang , S. Huang , ACS Omega 2019, 4, 18771. 31737838 10.1021/acsomega.9b02734 PMC6854835

[52] L. Novotny , B. Hecht , Principles of Nano‐Optics, Cambridge University Press, Cambridge 2006.

[53] R. O. Prum , R. H. Torres , S. Williamson , J. Dyck , Nature 1998, 396, 28.

[54] W. Man , M. Florescu , E. P. Williamson , Y. He , S. R. Hashemizad , B. Y. Leung , D. R. Liner , S. Torquato , P. M. Chaikin , P. J. Steinhardt , Proc. Natl. Acad. Sci. U. S. A. 2013, 110, 15886. 24043795 10.1073/pnas.1307879110 PMC3791749

[55] S. F. Liew , J. K. Yang , H. Noh , C. F. Schreck , E. R. Dufresne , C. S. O'Hern , H. Cao , Phys. Rev. A 2011, 84, 6.

[56] D. Weaire , Phys. Rev. Lett. 1971, 26, 1541.

[57] R. Vaz , M. F. Frasco , M. G. F. Sales , Nanoscale Adv. 2020, 2, 5106. 10.1039/d0na00445f PMC9416915 36132040

[58] S. Tadepalli , J. M. Slocik , M. K. Gupta , R. R. Naik , S. Singamaneni , Chem. Rev. 2017, 117, 12705. 28937748 10.1021/acs.chemrev.7b00153

[59] A. E. Seago , R. Oberprieler , V. K. Saranathan , Int. Comp. Biol. 2019, 59, 1664. 10.1093/icb/icz040 31093648

[60] B. D. Wilts , P. L. Clode , N. H. Patel , G. E. Schröder‐Turk , MRS Bull. 2019, 44, 106.

[61] J. Schindelin , I. Arganda‐Carreras , E. Frise , V. Kaynig , M. Longair , T. Pietzsch , S. Preibisch , C. Rueden , S. Saalfeld , B. Schmid , J. Y. Tinevez , D. J. White , V. Hartenstein , K. Eliceiri , P. Tomancak , A. Cardona , Nat. Methods 2012, 9, 676. 22743772 10.1038/nmeth.2019 PMC3855844

[62] P. Thévenaz , U. E. Ruttimann , M. Unser , IEEE Trans. Image Process. 1998, 7, 27. 18267377 10.1109/83.650848

[63] A. Parslow , A. Cardona , R. J. Bryson‐Richardson , J. Vis. Exp. 2014, 86, e51086. 10.3791/51086 PMC4166950 24747942

[64] I. Arganda‐Carreras , V. Kaynig , C. Rueden , K. W. Eliceiri , J. Schindelin , A. Cardona , H. S. Seung , Bioinformatics 2017, 33, 2424. 28369169 10.1093/bioinformatics/btx180

[65] M. Wohlgemuth , N. Yufa , J. Hoffman , E. L. Thomas , Macromolecules 2001, 34, 6083.

