# Supplementary material for: 3D Tomographic Analysis of the Order‐Disorder Interplay in the Pachyrhynchus congestus mirabilis Weevil
Source: Adv Sci (Weinh). 2022 Jul 18;9(26):2202145. doi: 10.1002/advs.202202145 (PMC9475527; doi:10.1002/advs.202202145)
Supplement: Supplementary file 1 — Supporting Information [file ADVS-9-2202145-s001.pdf]

ADVANCED SCIENCE
Open Access

Supporting Information

for *Adv. Sci.*, DOI 10.1002/advs.202202145

3D Tomographic Analysis of the Order-Disorder Interplay in the *Pachyrhynchus congestus mirabilis* Weevil

*Kenza Djeghdi, Ullrich Steiner and Bodo D. Wilts**

Supporting Information
for
3D tomographic analysis of the order-disorder interplay in the *Pachyrhynchus congestus mirabilis* weevil

Kenza Djeghdi[1], Ullrich Steiner[1], and Bodo D. Wilts[1,2,*]

[1]Adolphe Merkle Institute, University of Fribourg, Chemin des Verdiers 4, 1700 Fribourg, Switzerland
[2]Chemistry and Physics of Materials, University of Salzburg, Jakob-Haringer-Straße 2a, 5020 Salzburg, Austria
[*]Corresponding author, e-mail: bodo.wilts@plus.ac.at

Imaging infiltrated scales

Cross-sections of scales (Fig. S1) were taken by FIB-SEM after in-situ Pt filling and showed a strong contrast between the two phases of the bicontinuous networks when using the T1 detector.

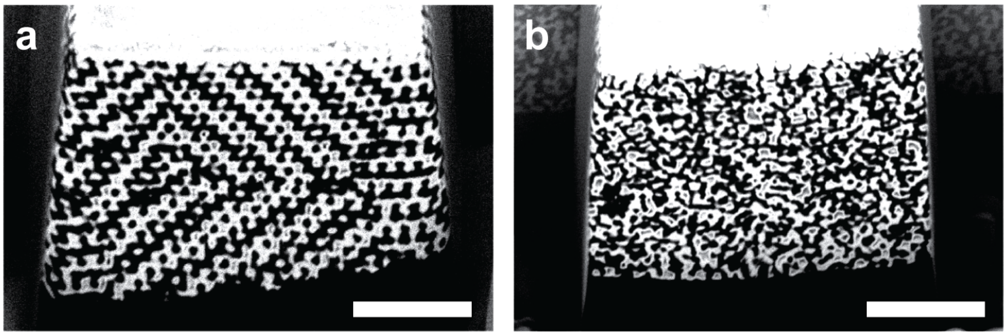

Figure S1: SEM image post Pt infiltration of a) an ordered red scale and b) a disordered blue scale. The chitin phase appears in black and the more conductive Pt phase appears in white. Scale bars: 2 µm.

Angle definitions

A bond angle is the angle formed between three atoms across two bonds. It is defined by

$$\theta = \cos^{-1}\left(\frac{\vec{r_1} \cdot \vec{r_2}}{\|\vec{r_1}\| \cdot \|\vec{r_2}\|}\right).$$

A (proper) dihedral angle (also called torsion angle) is defined as the angle between two planes which are delimited by two bonds emerging from neighboring atoms,

$$\phi = \mathrm{atan2}(\vec{r_2} \cdot ((\vec{r_1} \times \vec{r_2}) \times (\vec{r_2} \times \vec{r_3})), \|\vec{r_2}\|(\vec{r_1} \times \vec{r_2}) \cdot (\vec{r_2} \times \vec{r_3})).$$

Representation of both angles is shown Fig. S2.

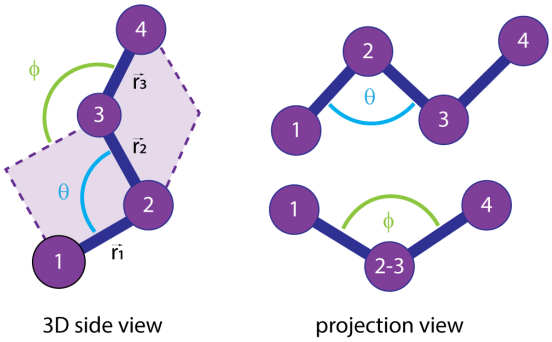

Figure S2: Definition of bond angle θ (blue) and dihedral angle ϕ (green).

Transmission spectra

Transmission spectra (Fig. S3) were taken in refractive index matching fluid to eliminate the contribution of structural coloration, thereby investigating the presence of pigments. The refractive index was calculated from the Cauchy law [1] and the absorption coefficient was calculated from the transmission spectra using

$$\mathrm{Re}(n_{\mathrm{melC}}) = 1.517 + \frac{8800}{\lambda^2}, \tag{1}$$

$$\mathrm{Im}(n_{\mathrm{melC}}) = -\log_{10}(\mathrm{T})\ \frac{\lambda}{4\pi \mathrm{d} \log_{10}(\mathrm{e})}, \tag{2}$$

where T is the transmission value, λ is the wavelength, and d is the thickness of the scale.

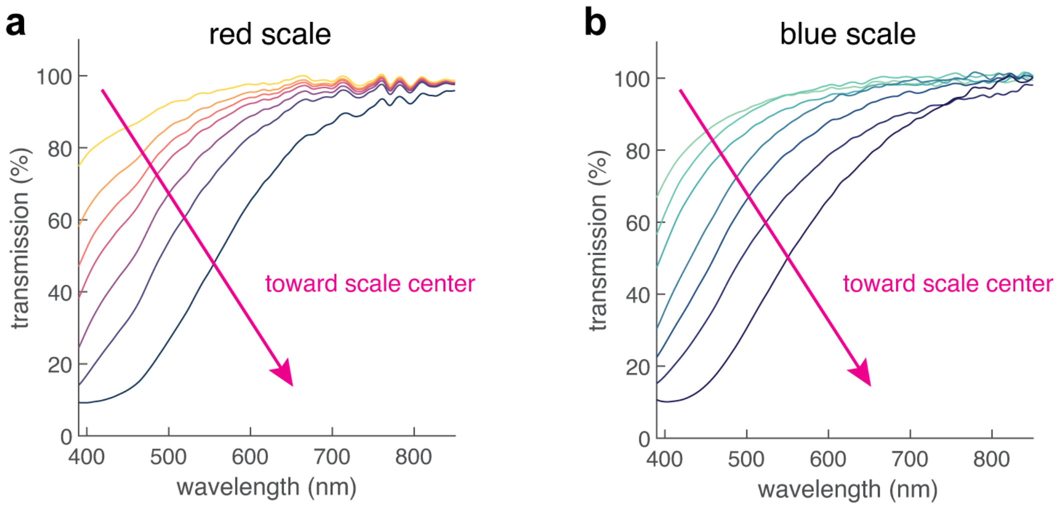

Figure S3: Transmission spectra taken at various positions of a red (a) and a blue (b) scale immersed in a fluid matching the refractive index of chitin ($n = 1$ for each curve). The amount of pigment increases from the edge of the scale to the stem, giving rise to an absorption gradient. The curves have been smoothed to remove experimental noise.

3D representation

Additional representations of the reconstructed sub-volumes are shown in Fig. S4. In this representation, the plane imaged by the SEM is $x - y$, the ion beam is aligned with the $O - x$ axis and the slicing direction is along the $O - z$ axis.

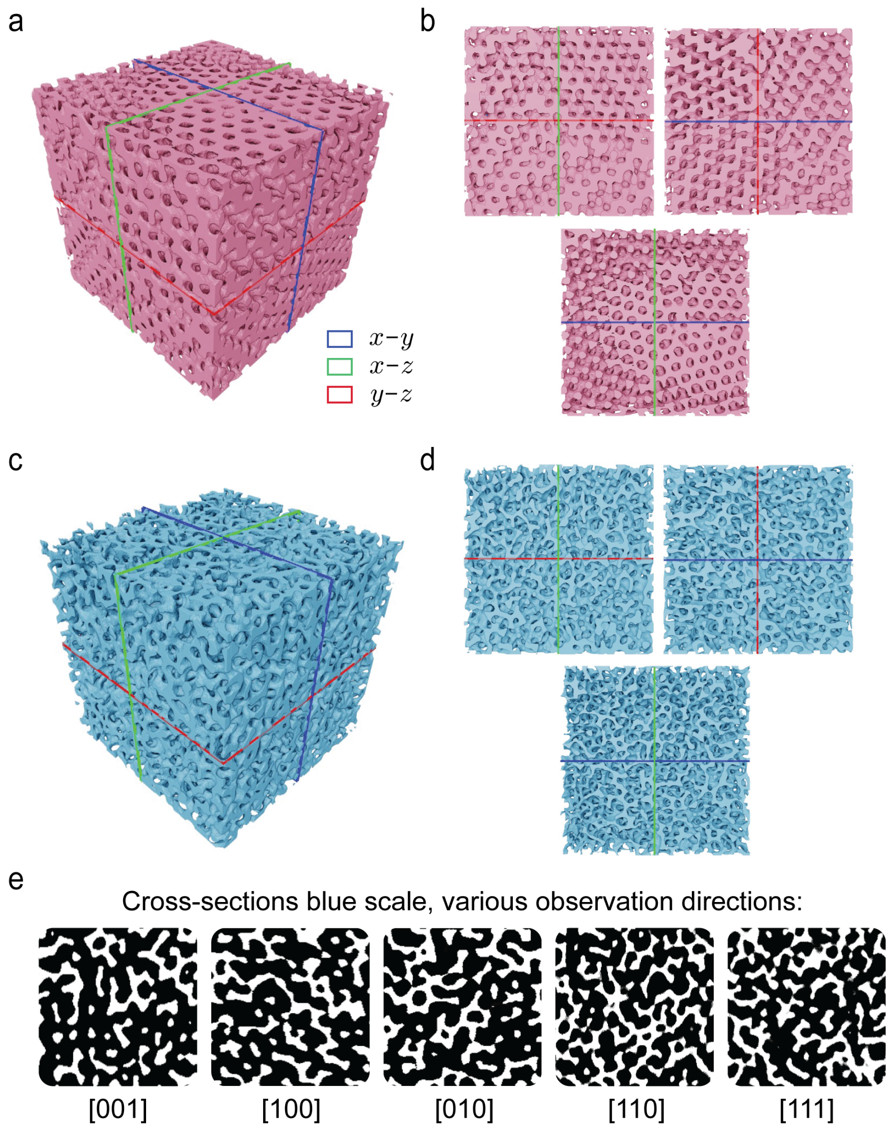

Figure S4: Sub-volume $V = (3.6\,\mu\text{m})^3$ of the a) red and c) blue scales. $x - y$, $x - z$, and $y - z$ planes of the b) red and d) blue scales. e) Cross-sections through the 3D dataset of the blue scale. The Miller indices correspond to the direction of observation in the referential defined above.

References

[1] D. G. Stavenga, H. L. Leertouwer, T. Hariyama, H. A. De Raedt, B. D. Wilts, PloS one **2012**, 7, 11 e49743.
